# Analysis of the Toll-Like Receptor 2-2 (TLR2-2) and TLR4 mRNA Expression in the Intestinal Mucosal Immunity of Broilers Fed on Diets Supplemented with Nickel Chloride

**DOI:** 10.3390/ijerph110100657

**Published:** 2014-01-03

**Authors:** Bangyuan Wu, Hengmin Cui, Xi Peng, Jing Fang, Zhicai Zuo, Junliang Deng, Jianying Huang

**Affiliations:** Key Laboratory of Animal Diseases and Environmental Hazards of Sichuan Province, College of Veterinary Medicine, Sichuan Agricultural University, Ya’an 625014, China; E-Mails: wubangyuan2008@163.com (B.W.); pengxi197313@163.com (X.P.); fangjing4109@163.com (J.F.); zzcjl@126.com (Z.Z.); dengjl213@126.com (J.D.); hjy19860316@163.com (J.H.)

**Keywords:** nickel chloride, TLR2-2, TLR4, mRNA expression, intestinal mucosal immunity, broilers

## Abstract

Toll-like receptor (TLRs) are important innate immune receptors, and TLR2 and TLR4 play an important role in intestinal mucosal innate immunity. It has been found that nickel (Ni) can affect the immune system in broilers. The purpose of this study was to analyze changes in TLR2-2 and TLR4 mRNA expression levels in the intestinal mucosal immunity system of broilers induced by dietary nickel chloride (NiCl_2_) using quantitative real-time polymerase chain reaction (qRT-PCR) assays. Two hundred and forty one-day-old avian broilers were divided into four groups and fed on a corn-soybean basal diet as control diet or the same basal diet supplemented with 300, 600 and 900 mg/kg of NiCl_2_ for 42 days. Results showed that the TLR2-2 and TLR4 mRNA expression levels in the intestinal mucosa and the cecal tonsil were lower (*p* < 0.05 or *p* < 0.01) in the 300, 600 and 900 mg/kg groups than those in the control group. It was concluded that dietary NiCl_2_ in excess of 300 mg/kg could reduce TLR2-2 and TLR4 mRNA expression levels in the intestinal mucosa and cecal tonsil in broilers, implying that the innate immunity in intestinal mucosal immune system could be impaired by pathways involving injured surface epithelium cells or/and the inhibition of the TLR signal transduction.

## 1. Introduction

Nickel (Ni) is used in a wide variety of industrial and consumer applications [1], and it is known to be an essential element for animals [2,3]. As an important metal pollutant, Ni is of considerable concern because its concentration is rapidly increasing in soils of different parts of the World and it is ultimately taken up by plants. Thus, Ni or Ni compounds can enter the food chain and cause deleterious effects on animals [4]. Human exposure to Ni metal and/or Ni oxide may occur via the main exposure routes (inhalation, ingestion or dermal contact) at occupational settings. Exposure to Ni metal or Ni- containing alloys can also occur via coins [5,6,7]. Ionic Ni (Ni^2+^) is thought to be the actual carcinogenic species because it can bind to cellular components, including nuclear proteins and DNA [8]. Ni complexes with heterochromatin lead to manifold alterations such as condensation, DNA hypermethylation and gene slicing, which disturb gene expression [5,9,10]. As a toxic metal, Ni is responsible for both severe and health effects, allergic contact dermatitis and respiratory tract cancer [11]. Anke *et al*. studied the effects of dietary nickel on laying hens [12]. Ling and Leach also reported that diets supplemented with from 125 to 250 mg/kg nickel had no effect on feed consumption or egg production, and above 300 mg/kg, nickel has been shown to be toxic to 3-week-old male chicks [13]. Furthermore, research has clearly identified that Ni can induce the innate immune response and interact with the signal transduction of TLRs [14].

TLRs are a family of membrane proteins that play an important role in host defenses against microbial pathogens by triggering the innate immune response and by inducing signals that initiate an adaptive immune response [15]. Consistent with their roles in immune surveillance, TLR mRNA are expressed at higher levels in tissues and cells such as the lungs, gastrointestinal tract, spleen [16], several small intestinal epithelial cells, colon, gastric pit cells, fetal intestinal cells and so on [17]. Furthermore, numerous pro- and anti-inflammatory cytokines are produced in response to bacterial ligands via activation of TLRs, for example, IL-1, IL-6, IL-8, IL-10, IL-12, IFN and TNF-α [18,19,20,21,22]. In addition to the immune functions, invertebrate TLRs have been shown to act during development and in cell to cell interactions [23,24,25]. Further investigations have shown that some TLRs act as coreceptors can promote or inhibit cellular responsiveness to activating ligands [26,27]. TLR2 and TLR4 have been shown to play critical roles in the pattern recognition of the surface components of bacteria and yeasts and in the initiation of the innate immune responses to these microbes [28].

The intestine is an important component of the mucosal immune system and plays an important role in host defense. The cecal tonsil is an important component in the avian mucosal immune system and provides important and unique immune functions. The cecal tonsil produces antibodies, functions as a secondary lymphoid organ, and has a sentinel role [17] and more diffuse lymphoid tissue and unorganized lymphoid follicles are present both in the mucosa and submucosa of the cecal tonsil in the broiler [29]. The intestinal immunity can be divided into innate and specific (adaptive) defenses [30]. The innate immune system recognizes pathogens by means of certain conserved structural features (also called pathogen-associated molecular patterns) of the microbes such as lipopolysaccharides (LPSs), major components of the outer membrane of Gram-negative bacteria [31,32]. It is possible that bacterial products penetrate the epithelial barrier, either due to damage or via paracellular pathways, to directly stimulate the underlying constituents of the mucosal immune system [33]. A variety of studies have provided increasing evidence that the surface epithelium serves a critical function as the defensive front line of the mucosal innate immune system in the gastrointestinal tract [34]. The intestinal epithelial cells can produce a marked effect in the intestinal mucosal immune system, and influence antigen presentation to LPL, even without direct contact [35]. Also, it has been proven that various intestinal epithelial cell lines constitutively express several members of a novel family of transmembrane receptors designated Toll-Like Receptors (TLRs) which may serve as a major link between innate and adaptive mucosal immune responses [36].

The gastrointestinal tract is one of the main ways that metals (including Ni) are absorbed [37], since the tract is exposed to the highest concentrations of metals due to the daily food or water consumption. Some studies in experimental animals and humans have shown that Ni intake causes immune toxicity [5,38] and dietary nickel chloride (NiCl_2_) induces intestinal oxidative damage, inhibits the intestinal development and decreases the serum cytokine contents in broilers [39,40,41].

In view of the abovementioned references, there have been no studies on the effects of Ni or Ni compounds on the TLR2 (TLR2-2) and TLR4 mRNA expression levels in the intestinal mucosa of animals and human so far. The aims of the present study were therefore to investigate the changes of TLR2 and TLR4 mRNA expression levels in the intestinal mucosa and the cecal tonsil in broilers induced by dietary NiCl_2_ using quantitative real-time polymerase chain reaction (qRT-PCR) assays, and to provide new experimental evidences for understanding the mechanism of the effects of NiCl_2_ or Ni on the innate immune responses.

## 2. Materials and Methods

### 2.1. Chickens and Diets

Two hundred and forty one-day-old healthy avian broilers were randomly divided into four groups with 60 broilers in each group. Broilers were housed in cages with electrically heated units and were provided with water as well as undermentioned experimental diets *ad libitum* for 42 days. A corn-soybean basal diet formulated by the National Research Council [42] was the control diet. NiCl_2_·6H_2_O was mixed into the corn-soybean basal diet to produce experimental diets with 300, 600 and 900 mg/kg of NiCl_2_, respectively.

### 2.2. Detection of TLR2 and TLR4 mRNA Expression Levels in the Intestinal Mucosa and the Cecal Tonsil by qRT-PCR

At 14, 28, and 42 days of age during the experiment, five broilers in each group were humanely killed and the intestinal tract were immediately removed and chilled to 0 °C in 0.85% sodium chloride (NaCl) solution, and the small intestine was divided into duodenum, jejunum and ileum. An approximately 4 cm intestinal segment was collected from the middle section of each intestinal region, and then was dissected and thoroughly cleaned with 0.85% NaCl solution. The mucosa was carefully scraped from the luminal face of the taken intestinal segments and stored in liquid nitrogen prior to the measurement. The cecal tonsils from the same five broilers in each group were also stored in liquid nitrogen for measurement.

The duodenal, jejunal and ileac mucosa and the cecal tonsils were crushed with liquid nitrogen by pestle until they turned into a homogeneous powder. Total RNA was extracted from the powder of the mucosa and the cecal tonsils using RNAiso Plus (9108/9109, Takara, Kyoto, Japan). The mRNA was then reverse transcribed into cDNA using PrimScript^TM^ RT reagent Kit with gDNA Eraser (RR047A, Takara) [43]. The cDNA was used as a template for quantitative real-time PCR analysis. Sequences for primers of TLR2-2 and TLR4 were obtained from Genbank and NCBI. Primers were designed using Primer 5 and synthesized at BGI Tech (Shenzhen, China), as shown in Table 1.

**Table 1:** A list of oligonucleotides used as primers in qRT-PCR analysis of mRNA expression in the intestinal mucosa and the cecal tonsil.

Gene Symbol	Accession Number	Primer	Primer Sequence (5′ → 3′)
TLR2-2	AB046533	F	AGGCACTTGAGATGGAGCAC
		R	CCTGTTATGGGCCAGGTTTA
TLR4	AY064697	F	AGTCTGAAATTGCTGAGCTCAAAT
		R	GCGACGTTAAGCCATGGAAG
β-Actin	L08165	F	TGCTGTGTTCCCATCTATCG
		R	TTGGTGACAATACCGTGTTCA

For qRT-PCR reactions, 25 µL mixtures were made by using SYBR^®^ Premix Ex Taq^TM^ II (DRR820A, Takara), containing 12.5 µL Tli RNaseH Plus, 1.0 µL of forward and 1.0 µL of reverse primer, 8.5 µL RNAase-free water and 2 µL cDNA. Reaction conditions were set to 3 min at 95 °C (first segment, one cycle), 10 s at 95 °C and 30 s at Tm of a specific primer pair (second segment, 44 cycles) followed by 10 s at 95 °C, and 72 °C for 10 s (dissociation curve segment) using Thermal Cycler (C1000, BioRad, Hercules, CA, USA). Gene expression was analyzed for 2 genes, and β-Actin was used as an internal control gene. Gene expression values at 14, 28 and 42 days of age were used for gene expression calibration, respectively. With 2^−ΔΔCT^ assay, the results were analyzed.

### 2.3. Statistical Analysis

Data of the control and three NiCl_2_ groups were statistically evaluated with the SPSS/16.0 software package for Windows. Hypothesis testing methods included one way analysis of variance (ANOVA) followed by least significant difference test. *p* < 0.05 was considered as statistical significance. All results were expressed as means ± standard deviation (*x* ± SD), representing five broilers in each group.

## 3. Results

### 3.1. Changes of the TLR2-2 mRNA Expression Levels

**Figure 1:**
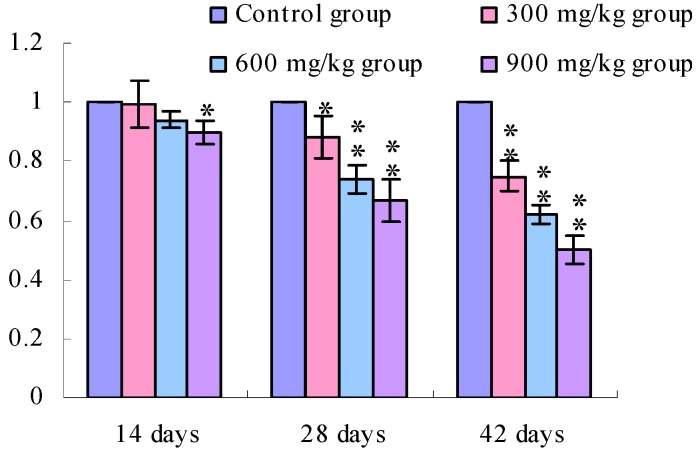
The TLR2-2 mRNA expression levels in the duodenal mucosa in broilers.

Note: Data are presented with the means ± standard deviation (*n* = 5); * *p* < 0.05, compared with the control group; ** *p* < 0.01, compared with the control group.

**Figure 2:**
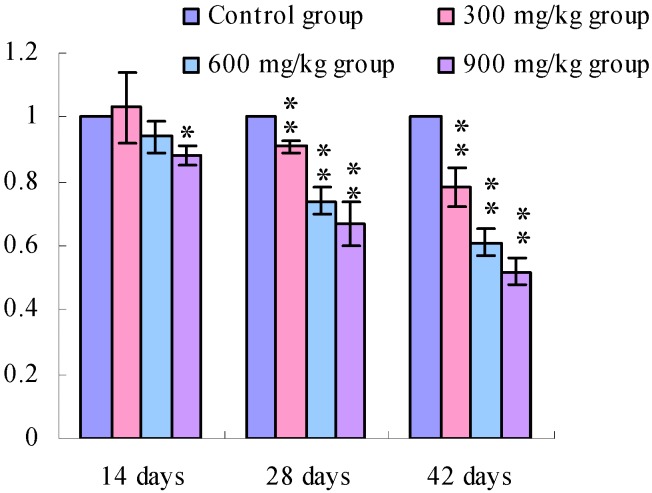
The TLR2-2 mRNA expression levels in the jejunal mucosa in broiler.

Note: Data are presented with the means ± standard deviation (*n* = 5); * *p* < 0.05, compared with the control group; ** *p* < 0.01, compared with the control group.

**Figure 3:**
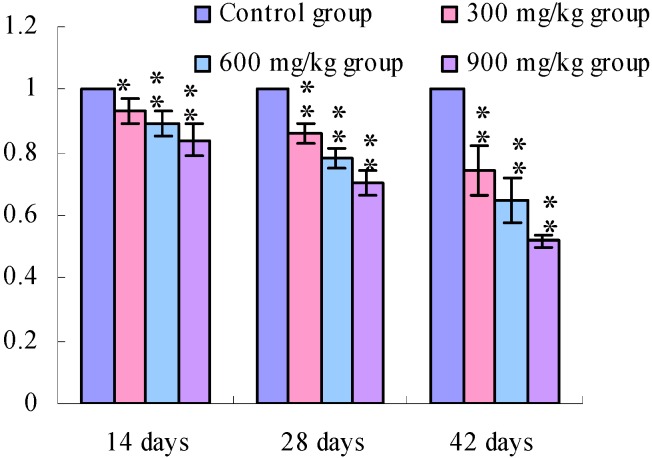
The TLR2-2 mRNA expression levels in the ileac mucosa in broilers.

Note: Data are presented with the means ± standard deviation (*n* = 5); * *p* < 0.05, compared with the control group; ** *p* < 0.01, compared with the control group.

**Figure 4:**
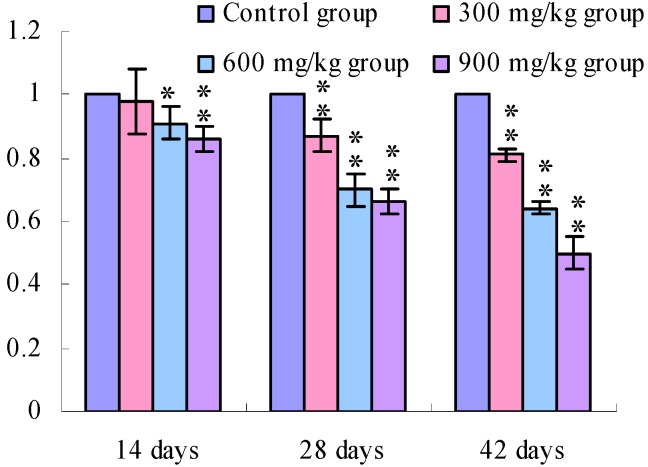
The TLR2-2 mRNA expression levels in the cecal tonsil in broilers.

Note: Data are presented with the means ± standard deviation (*n* = 5); * *p* < 0.05, compared with the control group; ** *p* < 0.01, compared with the control group.

Figure 1, Figure 2, Figure 3 and Figure 4 show that the TLR2 mRNA expression levels in the duodenum and jejunum were significantly decreased (*p* < 0.05) in the 900 mg/kg group at 14 days of age and were significantly decreased (*p* < 0.05 or *p* < 0.01) in the 300, 600 and 900 mg/kg groups when compared with those of the control group at 28 days of age and 42 days of age. The TLR2 mRNA expression levels in the ileum were significantly decreased (*p* < 0.05 or *p* < 0.01) in the 300, 600 and 900 mg/kg groups from 14 days of age to 42 days of age.

The TLR2 mRNA expression levels in the cecal tonsil were lower (*p* < 0.05 or *p* < 0.01) in the 600 and 900 mg/kg groups at 14 days of age and were significantly lower (*p* < 0.01) in the 300 mg/kg, 600 mg/kg and 900 mg/kg groups at 28 days of age and 42 days of age than those in the control group.

### 3.2. Changes of the TLR4 mRNA Expression Levels

**Figure 5:**
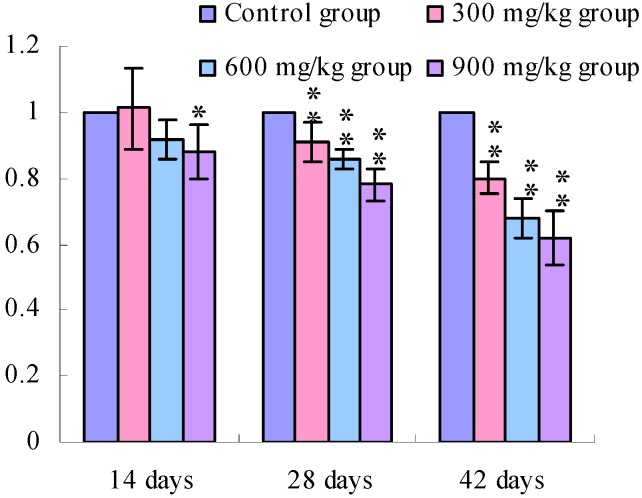
The TLR4 mRNA expression levels in the duodenal mucosa in broilers.

Note: Data are presented with the means ± standard deviation (*n* = 5); * *p* < 0.05, compared with the control group; ** *p* < 0.01, compared with the control group.

**Figure 6:**
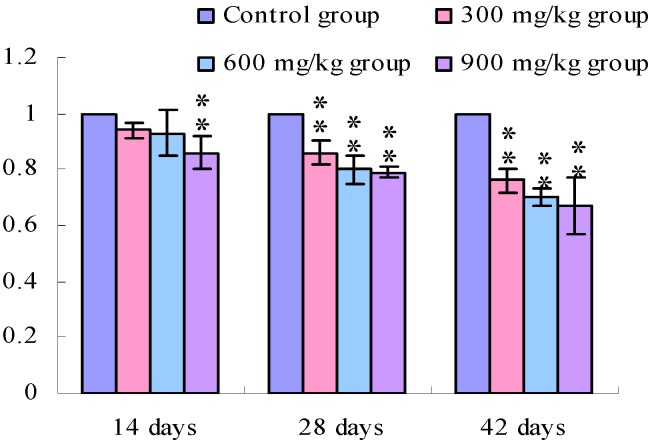
The TLR4 mRNA expression levels in the jejunal mucosa in broilers.

Note: Data are presented with the means ± standard deviation (*n* = 5); * *p* < 0.05, compared with the control group; ** *p* < 0.01, compared with the control group.

**Figure 7:**
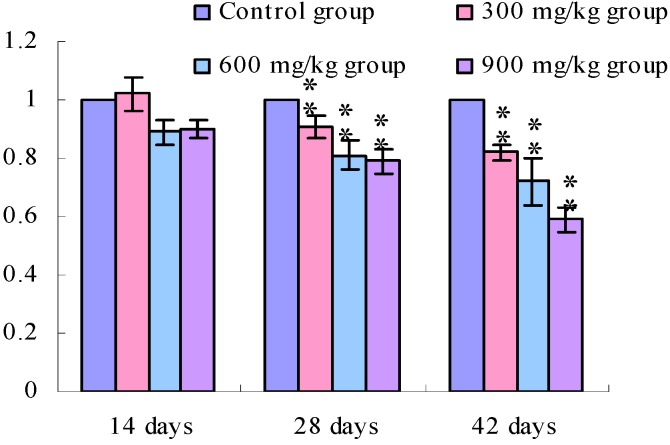
The TLR4 mRNA expression levels in the ileac mucosa in broilers.

Note: Data are presented with the means ± standard deviation (*n* = 5); * *p* < 0.05, compared with the control group; ** *p* < 0.01, compared with the control group.

**Figure 8:**
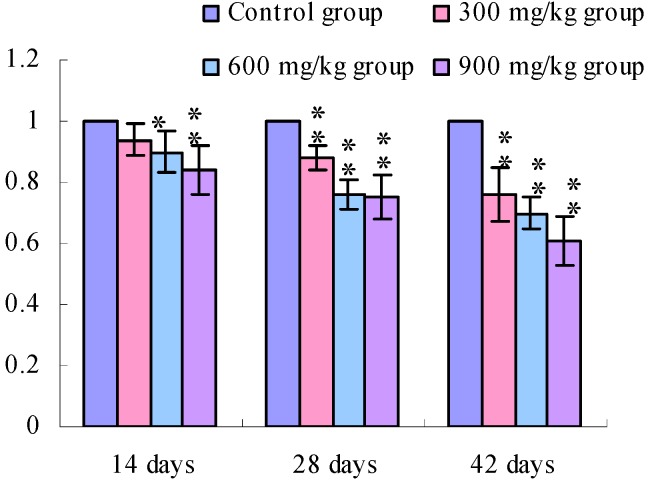
The TLR4 mRNA expression levels in the cecal tonsil in broilers.

Note: Data are presented with the means ± standard deviation (*n* = 5); * *p* < 0.05, compared with the control group; ** *p* < 0.01, compared with the control group.

The TLR4 mRNA expression levels in the duodenum and jejunum were significantly decreased (*p* < 0.05 or *p* < 0.01) in the 900 mg/kg group at 14 days of age and were significantly decreased (*p* < 0.01) in the 300, 600 and 900 mg/kg groups when compared with those of the control group at 28 days of age and 42 days of age. The TLR4 mRNA expression levels in the ileum were significantly decreased (*p* < 0.01) in the 300, 600 and 900 mg/kg groups at 28 days of age and 42 days of age. The TLR4 mRNA expression levels in the cecal tonsil were lower (*p* < 0.05 or *p* < 0.01) in the 600 and 900 mg/kg groups at 14 days of age and were significantly lower (*p* < 0.01) in the 300, 600 and 900 mg/kg groups from 28 to 42 days of age than those in the control group. The results are shown in Figure 5, Figure 6, Figure 7 and Figure 8.

## 4. Discussion

In the present study, TLR2-2 and TLR4 mRNA expression level changes induced by NiCl_2_ (Ni^2+^) were analyzed in the intestinal mucosa and cecal tonsil of broilers. It is reported that Ni^2+^ can directly trigger an innate immune response in resident skin cells that is necessary for mounting an allergic hypersensitivity reaction to Ni^2+^ [44], and Ni may interact with TLR signal transduction, a critical pathway involved in the sensing of pathogen-associated molecular patterns and the production of proinflammatory mediators, a process dependent on induction of NF-κB activation. NF-κB proteins are regulators of the immune, inflammatory, stress, proliferative and apoptotic responses of a cell to a very large number of different stimuli [15,45,46]. It seems that the down-regulation of the (TLR2-2) and TLR4 reflects the consequences of the interaction of intestinal cells (especially the epithelial cells) with NiCl_2_ and may be involved in mechanism of the effect of NiCl_2_ or Ni on the innate immune responses.

Upon recognition of microbes, dendritic cells (DCs)^4^ produce inflammatory cytokines that induce innate responses and up-regulate their costimulatory molecules to promote adaptive immunity [47]. Recognition of microbes is mediated by pattern recognition receptors (PRRs), including the TLR family [48]. In the chicken, TLR2 and TLR4 have been identified and characterised at the molecular and functional levels [49,50,51]. It is reported that TLR2 and TLR4 play important roles in the mucosal epithelial barrier function [52,53]. In the present study our attention was concentrated on the expression of TLR2 (TLR2-2) and TLR4 and its role in the innate immunity. Down-regulation of TLR2 (TLR2-2) and TLR4 mRNA expression levels in the intestinal mucosa and the cecal tonsil were observed in the 300, 600 and 900 mg/kg groups, indicating that the mucosal epithelial barrier function or immune function may be impaired by dietary NiCl_2_.

TLR2 as an important TLR that can control mucosal inflammation by regulating epithelial barrier function [52], and NiSO_4_ alters the pattern of TLR-2–dependent chemokine release from human lung fibroblasts (HLF) via a COX-2–mediated pathway [54]. TLR2 can also induce cell apoptosis through MyD88 via a pathway involving Fas-associated death domain protein (FADD) and caspase 8 [55]. The functional properties of type 2 TLR2 (TLR2-2) are similar to those of mouse and human TLR2 [50]. In the present study, TLR2-2 mRNA expression levels in the intestinal mucosa and the cecal tonsil were decreased in the 300, 600 and 900 mg/kg groups, which implied that dietary NiCl_2_ in excess of 300 mg/kg could impact the epithelial barrier function of the intestinal mucosa and decrease the ability of TLR2 in response to the antigens, and at last the innate immune system in the intestinal mucosa was impaired. Moreover, we found statistically significant down-regulation of TLR4 expression in the small intestine and the cecal tonsil in broilers. TLR4 has been genetically identified as an essential and nonredundant component of the LPS receptor signaling complex that controls innate immune responses *in vivo* [56,57,58]. TLR4 signaling has been divided into MyD88-dependent (responsible for proinflammatory cytokine expression) and MyD88-independent (TRIF-dependent) pathways (that mediate the induction of Type I interferons and interferon-inducible genes) [59,60]. LPS activates the MyD88/IRAK (IL-1 receptor-associated kinase) signaling cascade in monocytic and in endothelial cells [61]. Structurally, some findings suggest that Ni may directly bridge two adjacent TLR4 molecules, allowing a dimer formation similar to that induced by LPS, which is sufficient for signal transduction [62]. TLR4, as the receptor for the bacterial membrane component LPS, has been identified as the crucial mediator of the innate immune response to Ni^2+^, demonstrating that Ni^2+^ employs signaling components of the bacterial defense system to elicit its allergic reactions [44]. In the present study, TLR4 mRNA expression levels in the intestinal mucosa and the cecal tonsil were decreased in the 300, 600 and 900 mg/kg groups, demonstrating that dietary NiCl_2_ in excess of 300 mg/kg can impact the innate immune system in the intestinal mucosa, and it may related to the inhibition of the two signaling pathways of TLR4, and at last the signal transduction is inhibited by dietary NiCl_2_ (Ni^2+^).

Based on the results of our study, we can suggest that NiCl_2_ (Ni^2+^) can interact with TLR signal transduction through NF-κB activation, and it is reported that the common downstream signaling pathway of TLR2 and TLR4 leads to the activation of NF-kB through myeloid differentiation protein (MyD88) and IL-1 receptor-associated kinase in various cell types [63,64,65]. It seems that NF-κB activation or other related protein or receptors may be inhibited by NiCl_2_ (Ni^2+^). Furthermore, to our knowledge, the down-expression of TLR2-2 and TLR4 is possibility associated with the lesions of intestine and cecal tonsil and the cytokines secretion. Lesions in the small intestine and cecal tonsil induced by dietary NiCl_2_ have been observed in our recent research [39,40,66], including the oxidative stress and apoptosis induced by NiCl_2_. The lesions can cause decrease in epithelial cells or other cells in the small intestine and cecal tonsil, which leads down-regulation in the expression of TLR2-2 and TLR4. Meanwhile, the expression of TLR related to the cytokines. It was observed in the present study that dietary NiCl_2_ in excess of 300 mg/kg could inhibit certain cytokines secretion. Therefore, decreased cytokines contents could inhibit the activity of intestinal cells, and lead the expression of TLR2 and TLR4 down-regulation finally. Another pathway that is demonstrated to be influenced by Ni^2+^ is the death receptor-mediated or extrinsic apoptosis pathway [67] of the intestinal cells, which may related to the down-regulation in the expression of TLR2-2 and TLR4.

## 5. Conclusions

According to the results described in the present study and the aforementioned discussion, it is concluded that dietary NiCl_2_ in excess of 300 mg/kg reduces TLR2-2 and TLR4 mRNA expression levels in the intestinal mucosal and cecal tonsil, which indicates that the function of the defensive front line of the mucosal innate immune system in the intestine, and the innate mucosal immune responses may be impaired in broilers. Decrease in mRNA expression levels of TLR2-2 and TLR4 of intestinal mucosa (duodenum, jejunum and ileum) and cecal tonsil induced by NiCl_2_ is closely related to the injured surface epithelium cells or/and the inhibition of the TLR signal transduction. Further studies are required to reach a more defined elucidation of the mechanisms involved in these complex processes.
